# New records of the colonial chrysophyte *Urostipulosphaeraarticulata* (Chrysophyceae, Ochromonadales) in China

**DOI:** 10.3897/BDJ.13.e150900

**Published:** 2025-06-12

**Authors:** Yalu An, Junxue Hao, Fangru Nan, Junping Lv, Qi Liu, Xudong Liu, Shulian Xie, Jia Feng

**Affiliations:** 1 Shanxi University, Taiyuan, China Shanxi University Taiyuan China

**Keywords:** Chrysophyceae, morphology, phylogenetic studies, taxonomy, *Urostipulosphaera*

## Abstract

**Background:**

The genus *Urostipulosphera* was established in 2019. Its morphological characteristics are similar to *Uroglena* and *Uroglenopsis*, but it differs from both. This genus connects individual cells into communities through a dichotomous branching structure, which distinguishes it from *Uroglena* and *Uroglenopsis*. Although there are nine species in this genus, molecular data are available for only four species. Molecular data and species distribution records for *Urostipulosphaera* are severely lacking. Species of this genus have been recorded in a few locations in Europe and North America.

**New information:**

We identified three samples that were discovered in Shanxi Province, China, based on morphological characteristics and molecular phylogenetic analysis. Results of the polygenic phylogenetic tree, based on SSU, *rbc*L and ITS sequences, showed that the three samples were clustered with *Urostipulosphaeraarticulata* U5-5 from Czechia, with a high support rate of 100/1.00. Morphological observations further supported this result. Therefore, the three samples were identified as *Urostipulosphaeraarticulata*. This is the first report in China and enriches the geographic diversity of this species. Moreover, we found the long flagellum with mastigonemes under the scanning electron microscope complementing the description of the morphological characteristics of this species. The ITS2 secondary structure of the specimens differed from that of *Urostipulosphaeraarticulata* U5-5, which exhibited a loop and five-arm structure. A Bayesian relaxed clock analysis indicated that the genus *Urostipulosphaera* originated in the Early Cretaceous, approximately 109.85 million years ago (Ma) and diverged into two clades around 79.81 Ma.

## Introduction

The genus *Urostipulosphaera* was established by Pusztai and Škaloud (2019). The classification of its type species, *Urostipulosphaeranotabilis*, has gone through two revisions: initially, *Urostipulosphaeranotabilis* was classified as "*Uroglenanotabilis*" due to their dichotomous branching structures; later, Wujek and Thompson (2002) suggested that fine connecting lines may be a unique feature of *Uroglena* species and suggested the re-identification of *Urostipulosphaeranotabilis* as "*Uroglenopsisnotabilis*". Pusztai and Škaloud (2019) proposed a new genus *Urostipulosphaera* by combining morphological, ultrastructural and phylogenetic studies, clarifying the phylogenetic relationship amongst the three genera. Additionally, "*Uroglenopsisnotabilis*" was revised to *Urostipulosphaeranotabilis*. Meanwhile, they clarified that the key morphological differences amongst the three genera lie in the length ratio of unequal flagella and the connection structures between cells: *Urostipulosphaera* has two unequal flagella, with the longer flagellum approximately four times the length of the shorter one and its colonies are formed by gelatinous dichotomous branching structures at the cell posterior; *Uroglena* colonies are connected by thin threads formed at the cell posterior and the longer flagellum is approximately twice the length of the shorter one; *Uroglenopsis* cells are embedded in a tight jelly-like coat, lacking radiating cytoplasmic threads or gelatinous stems, with the longer flagellum approximately four times the length of the shorter one. Later, Pusztai and Škaloud (2022) reported a new species *U.granulata* and revised seven species (*U.articulata*, *U.lindiae*, *U.proxima*, *U.soniaca*, *U.conimamma*, *U.eustylis* and *U.europaea*) into this genus, based on *rbc*L + ITS molecular phylogeny. Of the nine species, only four species (*U.notabilis*, *U.granulata*, *U.articulata* and *U.lindiae*) were supported by molecular data (https://www.ncbi.nlm.nih.gov/) (accessed on 20 January 2025). There have been no further reports of this genus since then. The lack of molecular sequences of *Urostipulosphaera* species emphasises the necessity of utilising molecular markers for sequencing and supplementing molecular data.

We have described the morphological characteristics of the colonies, cells and stomatocysts of *Urostipulosphaeraarticulata* (SX230512, SX240513 and SX241121) collected from Shanxi Province in detail. Based on SSU, ITS and *rbc*L sequences, we conducted molecular phylogeny trees to confirm their taxonomic position and the phylogenetic relationships amongst *Urostipulosphaera* species and genera within Chrysophyceae. This paper aims to report the first geographic record of this rare species *U.articulata* in China and to enrich its geographical diversity, providing a resource for the study of the biodiversity of freshwater Chrysophyta in China. In addition, estimating the divergence time is of great significance for understanding the origin, evolution and adaptability of genera within Chrysophyceae.

## Materials and methods

### Sample collection and culture

**Figure 1.**
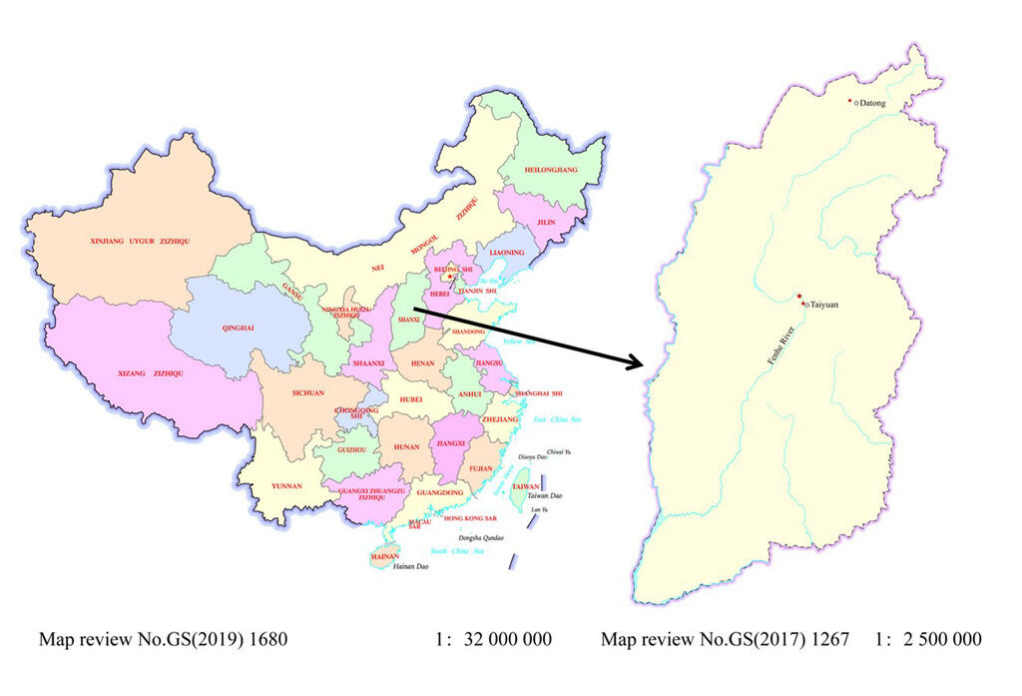
Map showing the localities where the samples were collected.

During 2023-2024, three samples (SX230512, SX240513 and SX241121) were collected from Taiyuan City and Datong City in Shanxi Province (Fig. 1). Sample SX230512 was collected on 12 May 2023 nearly the Xiangyun Bridge of Fenhe River (water temperature: 14.3℃, pH: 8.26). Sample SX240513 was collected on 13 May 2024 in the Jifen Bridge of Fenhe River (water temperature: 18.8℃, pH: 8.67). Sample SX241121 was collected on 13 May 2024 in the Wenyinghu Park in Datong City (water temperature: 4.8℃, pH: 8.22). All samples were collected using a plankton net with a mesh size of 20 μm. The colonies were isolated by pipetting; subsequently, washed colonies were placed individually into a cultured 24-well polypropylene plate, filled with DY-IV medium and sterilised raw water (1:1) at 14–16℃ under 12 h∶12 h light/dark cycle. The specimens were preserved with 4% formaldehyde. Voucher specimens were deposited in the Herbarium of Shanxi University (SXU), Shanxi University, Taiyuan, Shanxi Province, China.

### Morphological investigations

The morphological characteristics of the samples were observed and photographed under a BX-51 Olympus microscope (Olympus, Tokyo, Japan), equipped with a digital camera (DP72 Olympus, Tokyo, Japan). For SEM observations, 10 ml of algal fluid cultured to the logarithmic phase was placed in a centrifuge tube and centrifuged with a centrifuge set at 12000 r/min for 5 min. After centrifugation, the supernatant was discarded and transferred to the digestion tube and 2 ml nitric acid (HNO_3_) was added to the digestion instrument for digestion. The digestion temperature was set at 120℃ for 10 min. After cooling, the digested sample was transferred to a 10 ml centrifuge tube and distilled water was slowly added to 10 ml. The centrifuge was centrifuged at 8000 r/min for 10 min. The above operations were repeated seven times and the supernatant was discarded. A volume of 1 ml of 99% absolute ethanol was added to the centrifuged sample for preservation for subsequent electron microscopy. Finally, the prepared samples were placed on tin foil and fixed on a copper stage, critical-point dried and subjected to gold spraying for 1.5 min. The ultrastructure of stomatocysts was examined with a Hitachi Regulus 8100 (Tokyo, Japan) scanning electron microscope (SEM).

### Sequencing and phylogenetic analysis

We used a plant DNA extraction kit (Sangon Biotech, Shanghai, China) to extract DNA, from a harvested 1 ml of cultures in exponential growth by centrifugation for 5 min. The three molecular markers SSU, ITS and *rbc*L were amplified by polymerase chain reaction (PCR) to classify the species level within the Chrysophyceae. The PCR volume was 50 μl, consisting of 37.75 μl ddH_2_O, 5.0 μl 10× buffer, 4.0 μl 2.5 mM dNTPs, 0.25 μl Taq DNA polymerase (Sangon Biotech, Shanghai, China), 1.0 μl of each primer (10 mM) and 1.0 μl of genomic DNA. The reaction was undertaken in a MyCycler thermal cycler (Bio-Rad, Hercules, CA, USA). The amplification of SSU, ITS and *rbc*L used specific primers (Table 1). The SSU gene amplification programme was used as: initial denaturation at 94℃ for 5 min, followed by 35 cycles of 94℃ for 30 s, 47–54℃ for 30 s and 72℃ for 2 min, with a final extension step of 4℃ for 10 min. The ITS and *rbc*L genes were polymerase chain reaction-amplified with the following cycle conditions: 95℃ for 4 min; 35 cycles of 95℃ for 1 min, 41–48℃ for 1 min and 72℃ for 2 min and a final hold for 10 min at 4℃. The DNA sequences generated in this study have been deposited in GenBank under accession numbers (Suppl. material 1).

The newly-determined sequences were aligned to other sequences of Chrysophyceae from the GenBank database using MAFFT v. 7, applying the Q-INS-i strategy (Katoh et al. 2002). The sequences were selected according to Andersen et al. (2017) (Suppl. material 1). *Nannochloropsislimnetica* and *Synchromagrande* were chosen as outgroups. The final matrix contained 76 taxa (74 SSU rDNA, 19 ITS and 55 *rbc*L sequences). Using Bioedit v. 7.2.1 (Hall 1999), both ends of the sequences were cut to obtain an identical length alignment. According to the method of Zhang et al. (2020), we concatenated the SSU, ITS and *rbc*L sequences. Finally, the concatenated alignment consisted of 3780 bp, including 1722 bp of SSU rDNA, 1120 bp of ITS and 938 bp of *rbc*L. The appropriate model used the software PartitionFinder 2 to estimate, with all algorithms and AIC criterion, for BI: Subset (1) (2) (3) = GTR + I + G; for ML: Subset (1) (2) (3) = GTR + I + G (Lanfear et al. 2016). The IQ-TREE software was used to build a phylogenetic tree, based on Maximum Likelihood, with 5000 ultrafast bootstraps repetitions (Nguyen et al. 2015). MrBayes v. 3.2.6 software was used to build a Bayesian phylogenetic tree (Ronquist et al. 2012). MEGA 5.0 (Tamura et al. 2011) was used to calculate the pairwise p-distances of molecular sequences. Finally, the calculation results of each software were imported into FigTree 1.4.2 for editing. The final graphics were optimised using Adobe Illustrator software.

A Bayesian Inference method with a relaxed clock model was used to estimate branch divergence times using BEAST v. 2.0 (Drummond and Rambaut 2007) with the same three-genes dataset. The preferred approach to estimating the age of fossils was to use fossil-calibrated age estimates as probability priors (Drummond et al. 2006, Ho and Phillips 2009). The splits between *Mallomonasdenticulata* and M.striatavar.serrata, *M.foveata* and *M.elevata* were used for the lognormal priors. The three-genes concatenated dataset used a generalised time reversible (GTR) + gamma site model, with a uniform Yule tree before model speciation. All calibrations were based on the following setting: offset = 38 Ma, mean = 0.5 and standard deviation = 1.0. The analysis was run for 30 million generations with the chain sampled every 1000 generations. Tracer v. 1.7 (Rambaut et al. 2018) was used to check the convergence of parameter estimates and estimation of burn-in. The initial (10%) were removed as burn-in and the remaining trees were used to construct the final chronogram with 95% posterior probabilities (PP) and age estimates for all nodes. FigTree 1.4.2 was used to edit the resulting phylogenetic trees.

### ITS2 secondary structures

The boundaries of ITS1, 5.8S and ITS2 regions were determined by matching ITS sequences with the published *Synuraglabra* (access number: FM178511). The ITS2 secondary structures of the three samples were constructed using the fold computer programme. The consensus secondary structure was drawn using VARNA (Darty et al. 2009).

## Taxon treatments

### Urostipulosphaera articulata

(Korshikov) Pusztai & Skaloud, 2021

06B1BEBD-4FCB-5B9D-89A7-C45BCA3FE58B

https://www.ncbi.nlm.nih.gov/search/all/?term=Urostipulosphaera

#### Materials

- **Type status:** Other material. **Occurrence:** catalogNumber: SXU-SX230512; recordedBy: Zhao Kang-Xu; occurrenceID: C0FC4BFE-A7B0-5236-B3E6-A75A70A703A3; **Taxon:** scientificName: *Urostipulosphaeraarticulata*; class: Chrysophyceae; order: Ochromonadales; family: Ochromonadaceae; **Location:** country: China; stateProvince: Shanxi; county: Taiyuan; locality: the Fenhe River; verbatimCoordinates: 37.7743N 112.5378E; **Identification:** identifiedBy: An Ya-Lu; **Event:** year: 2023; month: 5; day: 12; **Record Level:** type: specimen; language: en; collectionCode: Algae
- **Type status:** Other material. **Occurrence:** catalogNumber: SXU-SX230512; recordedBy: Zhao Kang-Xu; occurrenceID: C0FC4BFE-A7B0-5236-B3E6-A75A70A703A3; **Taxon:** scientificName: *Urostipulosphaeraarticulata*; class: Chrysophyceae; order: Ochromonadales; family: Ochromonadaceae; **Location:** country: China; stateProvince: Shanxi; county: Taiyuan; locality: the Fenhe River; verbatimCoordinates: 37.7743N 112.5378E; **Identification:** identifiedBy: An Ya-Lu; **Event:** year: 2023; month: 5; day: 12; **Record Level:** type: specimen; language: en; collectionCode: Algae
- **Type status:** Other material. **Occurrence:** catalogNumber: SXU-SX230512; recordedBy: Zhao Kang-Xu; occurrenceID: C0FC4BFE-A7B0-5236-B3E6-A75A70A703A3; **Taxon:** scientificName: *Urostipulosphaeraarticulata*; class: Chrysophyceae; order: Ochromonadales; family: Ochromonadaceae; **Location:** country: China; stateProvince: Shanxi; county: Taiyuan; locality: the Fenhe River; verbatimCoordinates: 37.7743N 112.5378E; **Identification:** identifiedBy: An Ya-Lu; **Event:** year: 2023; month: 5; day: 12; **Record Level:** type: specimen; language: en; collectionCode: Algae

#### Description

**Figure 2.**
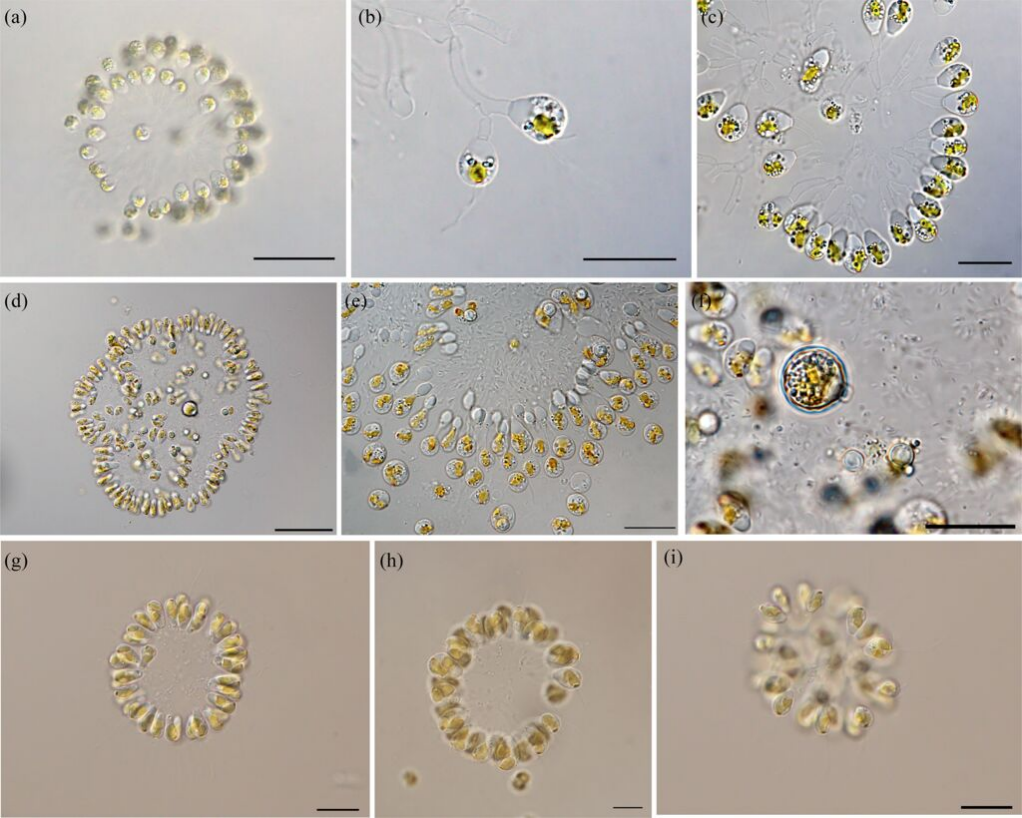
Morphology of *Urostipulosphaeraarticulata* from three localities in Shanxi Province light microscope (LM) images. **a-c** The sample of SX230512 morphological structures; **d-e** The sample of SX240513 morphological structures; **g-i** The sample of SX241121 morphological structures. Scale bar: a, d = 50 μm, b-c, e-i = 20 μm.

**Figure 3.**
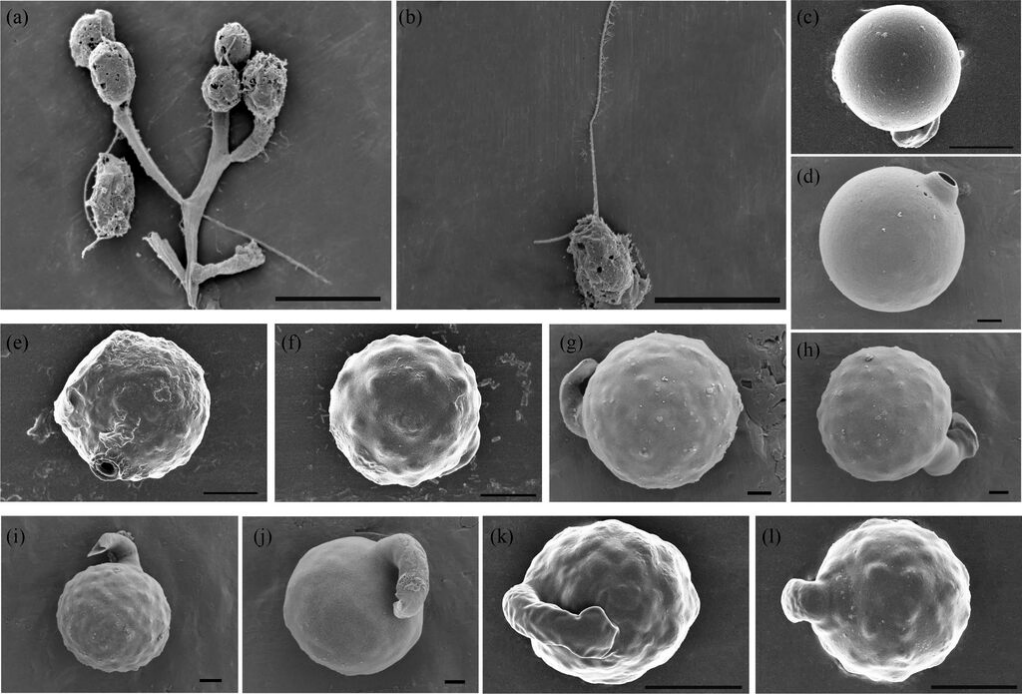
Morphological of cells and stomatocysts of *Urostipulosphaeraarticulata* from three localities in Shanxi Province (SEM). **a** Dichotomous branching structure; **b** Zoom on mastigonemes of long ﬂagellum. **c-e** immature stomatocysts. **d-l** immature stomatocysts. Scale bar: a, b, k, l = 10 μm, c, e, f = 5 μm, d = 3 μm, ,h, i, g, j = 2 μm.

The morphological colonies of *Urostipulosphaeraarticulata* from Shanxi Province were usually spherical, sometimes oval, cells posterior exhibited a truncate or rounded shape and they were connected into colonies via a dichotomous branching system of relatively thick gelatinous stalks (Fig. 2). The diameter of colonies ranged from 40–196 µm. Cells size varied from 10.8–18 µm long and 5.4–9 µm wide. Cells had a single, spiral, gold-coloured plastid with a red eyespot. The anterior end of the cell had two flagella of unequal length extending out of the cell. The longer flagellum ranges from 12.5 to 23.53 µm and the shorter one is between 3.6 µm and 5.5 µm, which is about 1/4 of the longer one. The long flagellum was with mastigonemes (Fig. 3b). Under the scanning electron microscope (SEM), the stomatocysts were found to be spherical, with diameters ranging from 12.5 to 15.5 μm. The mature cysts had a curved hook collar with irregular globular protrusions on the surface (Fig. 3g-l). In contrast, the immature cysts had a smooth surface and no complete collar (Fig. 3c-e).

The diameters of colony from sample SX230512 ranged from 40 to 120 µm and colonies consisted of tens to hundreds of obovate cells. Cell sizes varied from 10.8 to 18 µm long and 5.4 to 9 µm wide. The diameters of the mature cysts were 12.8–14.2 µm. The colony from sample SX240513 had a diameter of 83–196 µm, with cell dimensions of 13.09 to 14.29 µm x 5.95 to 7.14 µm. The cysts diameter were 12.56–13.77 µm. The diameter of colony from sample SX241121 was 52.63–67.85 µm and the cysts were 13.95–15.55 µm, the size of the cell being 11.90 to 13.09 µm long and 5.95 to 7.14 µm wide.

#### Distribution

Currently known from Germany, Russia, North America and Czechia.

## Analysis

### Phylogenetic analysis

**Figure 4.**
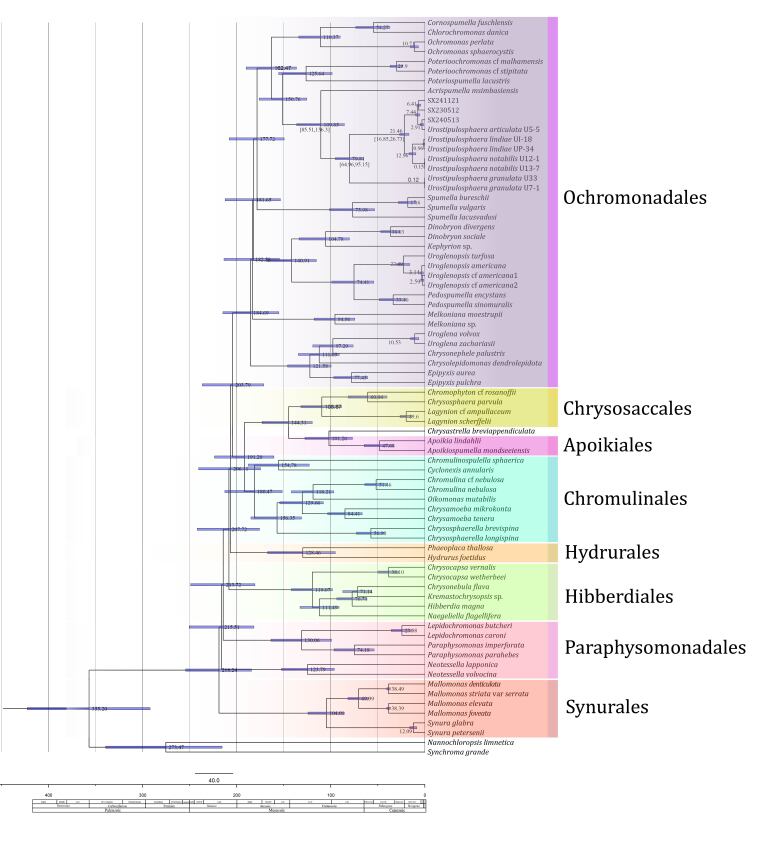
Maximum Likelihood tree based on concatenated SSU, ITS and *rbc*L sequences. Support values > 50% for all analyses are shown as follows: Maximum Likelihood bootstrap values (ML)/Bayesian posterior probabilities (BI). ‘-’ denotes < 50% support for that analysis at that node.

The pairwise distance and number of nucleotide variance of SSU, ITS and *rbc*L sequences amongst the taxa are shown in Suppl. materials 2, 3, 4. Based on SSU, ITS and *rbc*L sequences, two different conformational tree methods (Bayesian method and ML method) were used to construct phylogenetic trees (Fig. 4), revealing the taxonomic position of the three samples and the genus *Urostipulosphaera* within Chrysophyceae. The results showed that three samples were clustered together with *U.articulata* U5-5 from Czechia to form two branches. The samples SX230512 and SX241121 clustered with one branch with a high supporting value (99/1.00) and another branch consisted of the sample SX240513 and *U.articulata* U5-5 with a high support rate of 100/1.00. Additionally, the two branches had a high support rate of 100/1.00. The genus *Urostipulosphaera* was divided into two subclades (98/0.98). The first subclade only consisted of the two different strains of *Urostipulosphaeragranulata*. The second subclade consisted of *U.lindiae*, *U.notabilis* and *U.articulata*. *U.articulata* was closely related to *U.lindiae* and *U.notabilis* (100/1.00). The genus *Urostipulosphaera* formed a firmly supported monophyletic lineage within the Ochromonadales and is sister to *Acrispumellamsimbasiensis*. The *Uroglenopsis* was closely related to *Pedospumella* (92/1.00). *Ochromonasperlata* and *O.sphaerocystis* were closely related to *Chlorochromonas* and *Cornospumella* (100/1.00). *Uroglena* is sister to *Chrysonephelepalustris* with a support value of 99/0.99 and they were closely related to *Chrysolepidomonas* (90/0.97). Within Hibberdiales, *Hibberdia* was closely related to *Chrysonebula* and *Kremastochrysopsis*, with a support rate of 99/0.77. *Dinobryon* was closely related to *Kephyrion* (92/1.00). *Lagynion* was closely related to *Chrysophaera* and *Chromophyton* with a support value of 98/1.00. *Apoikia* and *Apoikiospumella* clustered together and the supporting value was as high as 100/1.00. *Chromulina* and *Oikomonasmutabilis* were closely related to *Chrysamoeba* (99/0.95). *Chrysosphaerellabrevispina* and *C.longispina* were clustered into one clade (100/1.00). *Lepidochromonas* and *Paraphysomonas* were clustered together with a high supporting value of 96/0.88.

**Figure 5.**
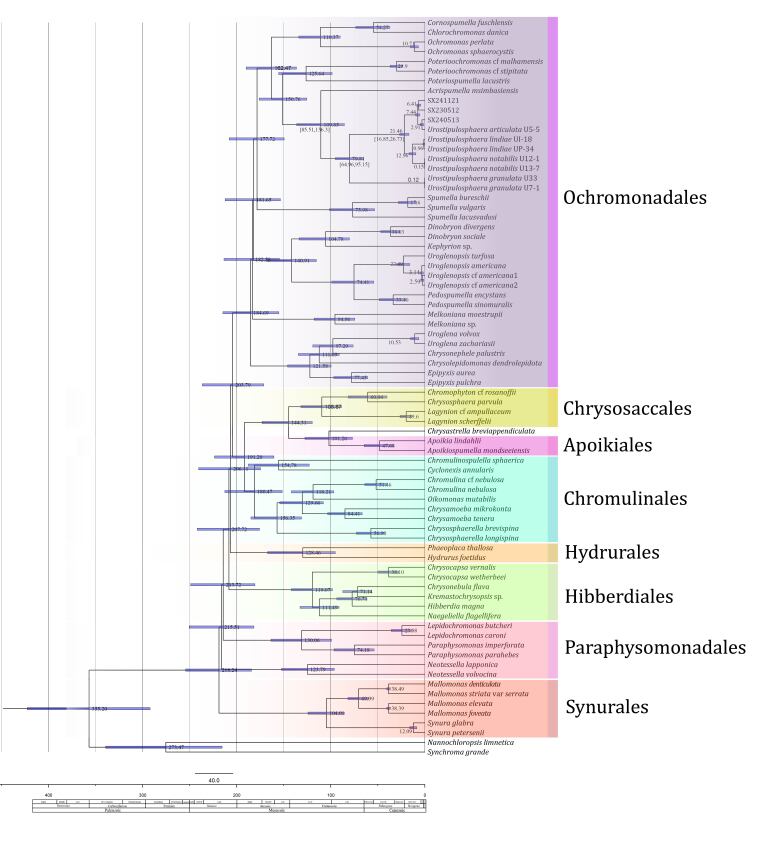
A timescale of the genus *Urostipulosphaera* from a Bayesian molecular clock analysis performed with BEAST.

Time-calibrated phylogenetic analyses estimated the divergence time of the genus *Urostipulosphaera* in Chrysophyta (Fig. 5). According to our estimations, the genus *Urostipulosphaera* most likely originated in the Early Cretaceous, approximately at 109.85 Ma. *Urostipulosphaera* split into two major subclades during the Late Cretaceous proximately at 79.81 Ma. *U.articulata* diverged from other *Urostipulosphaera* species between the Eocene Paleogene and Miocene Neogene, most likely approximately at 21.46 Ma (the Oligocene Paleogene).

### ITS2 secondary structures

**Figure 6.**
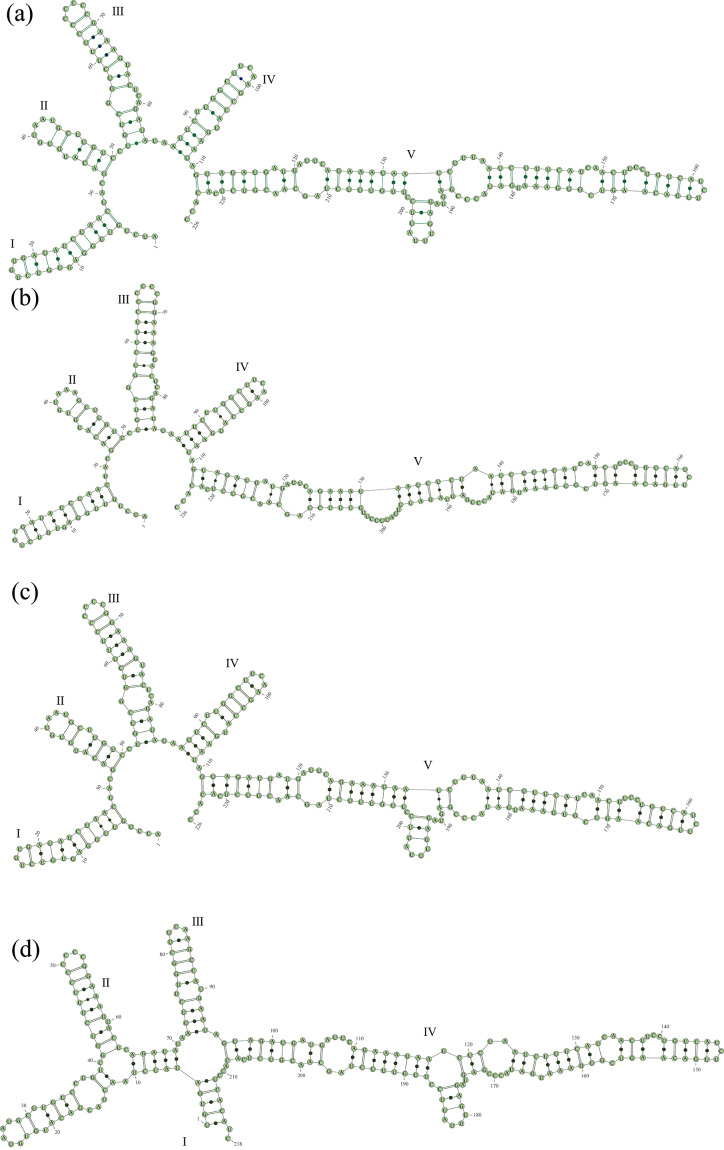
Predicted ITS2 secondary structures of the three samples *U.articulata* collected from Shanxi Province. **a** The secondary structures of sample of SX241121; **b** The secondary structures of sample of SX240513; **c** The secondary structures of sample of SX230512; **d** The secondary structures of strain of *U.articulata* 5-5.

We predicted the secondary structure of the three samples from different locations selecting *Synuraglabra* as a model and found that all of them had a one-ring and five-arm structure (Fig. 6a-c) fitting the model. The secondary structure of samples SX241121 and SX230512 highly overlapped and there were slight differences between them and SX240513 in the Ⅴ arms. However, they are quite different from *U.articulata* 5-5 (Fig. 6d) which is a ring with four arms. The base and pairing composition of ITS2 in *U.articulata* are shown in Table 2 and it can be found that the interspecies composition is similar. The overall A-U content was higher than the G-C content.

## Discussion

In general, chrysophytes typically have stringent environmental requirements and are predominantly found in cool, clean and nutrient-poor (oligotrophic) waters, exhibiting high sensitivity to temperature fluctuations. Combined with the Pusztai and Škaloud (2019) measured environmental factors and our documented result, we found that the living temperature range of *Urostipulosphaera* is relatively wide (0–18℃) and the water body is neutral or weakly alkaline. *U.granulata* is suitable for even lower temperatures close to zero. *U.articulata* has the largest temperature span in this genus and can be found in weakly alkaline water which allows it to survive in a variety of climatic and water conditions.

In our study, it was found that the three specimens were morphologically similar, but differed greatly in cell and colony size. The colonies from sample SX240513 had the largest diameter. The diameter of colony from samples SX230512 and SX241121 was greater than that described by Pusztai and Škaloud (2019) for *U.articulata* U5-5 and the diameter of colony from sample SX240513 was slightly smaller, but the three sample cells were elongated. We observed two flagella of unequal length at the anterior end, the shorter flagellum being approximately one-quarter the length of the longer one. Notably, when we observed three samples under the scanning electron microscope, we found that there was no scale structure, which is consistent with the previous Wujek (1969) finding. Furthermore, the surface of the cysts of the three samples collected from Shanxi Province differed from previous descriptions of *Urostipulosphaera*, the cysts of the samples being covered with globular bulges; the cysts of *U.articulata* U5-5 exhibited irregular bulges, the cysts of *U.granulata* and *U.notabilis* were covered with small granules and the cyst of *U.lindiae* displayed paw-like hooked processes. The mature cysts diameter of the samples was close to the *U.articulata* U5-5 between 12.8 and 15.55 μm. The immature cysts had a smooth surface and did not form a complete collar. Additionally, we did not observe a secondary collar, which was significantly different from *Uroglena*. Based on our observation using both light and electron microscopy, we proposed that the gelatinous dichotomous stalks may have been formed by the adhesion of residual parent cells during division (Fig. 2b and Fig. 3a), with individual cells of varying lengths of tubular structure adhering to each other to form a spherical community. Under the microscope, we observed that, in the sample SX230513, as the slide dried, the cells formed a thin line with an oil droplet trailing behind each cell (Fig. 2e). This thin line gradually elongated and thinned until it broke, causing the cells to detach from the oil droplet and eventually adopt a spherical shape. As the community disassembled, the edges of the cells became irregular and both the shape and number of plastids changed. The long flagellum fell off, while the shorter flagellum remained visible. Meanwhile, we found that heterokont flagella originate from an apical depression and the long flagellum with mastigonemes, while the short flagellum is naked (Fig. 3b). This is a new discovery of *Urostipulosphaera* that has not been described previously and provides important information for us to understand the morphological characteristics of this species. The motion characteristics of the samples were observed under the light microscope and it was found that the colonies moved forwards rotationally by the sway of the long flagellum, while the short flagellum did not seem to play an important role in the movement.

Furthermore, the phylogenetic tree, based on SSU, ITS and *rbc*L, provided strong molecular evidence that the samples SX230512, SX240513 and SX241121 are clustered with *U.articulata* with a high supporting value (100/1.00) and revealed the taxonomic position of the genus *Urostipulosphaera* in the Chrysophyceae that clustered with *Acrispumella* with a support rate of 83/0.90. In the phylogenetic trees, *U.lindiae* and *U.notabilis* clustered in a branch with a high support rate of 99/1.00 and *U.articulata* as a sister branch clustered with them (100/1.00), *U.granulata* clustering into a separate branch. However, in the Pusztai and Škaloud (2019) study, the result of the phylogenetic analysis was that *U.lindiae* and *U.articulata* clustered into a separate branch and *U.notabilis* as a sister branch clustered with them. Although *Uroglenopsis* and *Uroglena* had similar morphology to *Urostipulosphaera*, they were in different branches in the phylogenetic tree. *Uroglena* was clustered with *Chrysonephele* with a high support rate of 99/0.99 and *Uroglenopsis* formed a monophyletic lineage, sister to *Pedospumella* with a support rate of 92/1.00. This result supports the classification revision result of *Uroglena*-like by Pusztai and Škaloud (2019).

In addition, ITS2 is one of the most commonly used gene segments in phylogenetic analysis due to its conserved secondary structure, which can help assess species diversity within the genus (Boo et al. 2010, Kynclova et al. 2010, Škaloud et al. 2012, Škaloud et al. 2014). By predicting the secondary structure, we found that the secondary structure of three samples from Shanxi Province was a loop with five arms, while *U.articulata* 5-5 was a loop with four arms, but the base differences between the four strains were very small. By comparison, it was found that the reason for this phenomenon probably was due to base-pair substitutions, such as the appearance of G-U in the Ⅲ arms of the newly-collected specimens, which were not found in *U.articulata* 5-5.

The origin and evolution of the genus *Urostipulosphaera* have not been studied since its establishment in 2019. Based on the Bayesian relaxed clock analysis, the genus *Urostipulosphaera* and *Acrispumellamsimbasiensis* separated at about 109.85 Ma during the Early Cretaceous. Meanwhile, we discovered that the genus *Uroglenopsis*, which is morphologically similar to *Urostipulosphaera*, diverged around 74.41 Ma and the *Uroglena* diverged around 97 Ma, both later than *Urostipulosphaera*.

So far, molecular studies on chrysophytes are still limited to marker gene sequences such as SSU, ITS and *rbc*L and only seven genome sequences of the genera *Mallomonas*, *Synura*, *Neotessella*, *Ochromonas* and *Poterioochromonas* have been obtained (Ševčíková et al. 2015, Kim et al. 2019, Gastineau et al. 2021). Beisser et al. (2017) reported the transcriptome sequences of 18 chrysophyte strains, which was undoubtedly crucial for the subsequent research. It not only provided reference sequences for subsequent research, but also supplemented the transcriptome database. At present, there is still a lack of genome and transcriptome data for chrysophytes and we will strive to provide genome and transcriptome data for more species to enrich the database of Chrysophyceae.

## Supplementary Material

XML Treatment for Urostipulosphaera articulata

962A059A-2F90-5B13-8CB6-015853DDA962 10.3897/BDJ.13.e150900.suppl1 Supplementary material 1 Table S1 Data type Table Brief description Taxa and accession numbers used in this study. Newly-acquired strain is highlighted. File: oo_1345239.xlsx https://binary.pensoft.net/file/1345239 Yalu An, Junxue Hao, Fangru Nan, Junping Lv, Qi Liu, Xudong Liu, Shulian Xie and Jia Feng

36878777-3A05-530B-8D0C-FDAF19578F6B 10.3897/BDJ.13.e150900.suppl2 Supplementary material 2 Table S2 Data type Table Brief description Pairwise distance (lower-left matrix) and number of nucleotide variance (upper-right matrix) of SSU sequence amongst the taxa in this study. File: oo_1345297.xls https://binary.pensoft.net/file/1345297 Yalu An, Junxue Hao, Fangru Nan, Junping Lv, Qi Liu, Xudong Liu, Shulian Xie and Jia Feng

3FC5BFC9-59D8-5F15-AEDD-139CB59FA510 10.3897/BDJ.13.e150900.suppl3 Supplementary material 3 Table S3 Data type Table Brief description Pairwise distance (lower-left matrix) and number of nucleotide variance (upper-right matrix) of *rbc*L sequence amongst the taxa in this study. File: oo_1345289.xls https://binary.pensoft.net/file/1345289 Yalu An, Junxue Hao, Fangru Nan, Junping Lv, Qi Liu, Xudong Liu, Shulian Xie and Jia Feng

38BE5E47-8DBA-5E77-B0E9-417D261D2E72 10.3897/BDJ.13.e150900.suppl4 Supplementary material 4 Table S4 Data type Table Brief description Pairwise distance (lower-left matrix) and number of nucleotide variance (upper-right matrix) of ITS sequence amongst the taxa in this study. File: oo_1345270.xls https://binary.pensoft.net/file/1345270 Yalu An, Junxue Hao, Fangru Nan, Junping Lv, Qi Liu, Xudong Liu, Shulian Xie and Jia Feng

## Figures and Tables

**Table 1.** Primers for amplifying and sequencing of the nuclear SSU, ITS rDNA and the plastid *rbc*L gene.

Primer	Sequence (5’–3’)	References
SSU		(Katana et al. 2001)
18S-F	AACCTGGTTGATCCTGCCAGT	
18S-R	TGATCCTTCTGCAGGTTCACCTACG	
ITS		(Pusztai and Škaloud 2022)
Chryso_ITS_F	ATCATTTAGAGGAAGGTGA	
Chryso_ITS_R	GCTTCACTCGCCGTTACT	
*rbc*L		(Pusztai and Škaloud 2022)
Chryso_*rbc*L_F4	TGG ACD GAY TTA TTA ACD GC	
Chryso_*rbc*L_R7	CCW CCA CCR AAY TGT ARW A	

**Table 2.** Base ratio within the ITS2 genus *Urostipulosphaera*.

**Species**	**A**/%	**C**/%	**G**/%	**U**/%	**G+C**/%	**A+U**/%	**bp**
SX230512	26.11	21.68	18.14	34.07	39.82	60.18	226
SX240513	26.55	22.12	17.7	33.63	39.82	60.18	226
SX241121	26.11	22.12	18.14	33.63	40.27	59.73	226
*Urostipulosphaeraarticulata* U5-5	27.06	23.39	16.51	33.03	39.91	60.09	218
